# Brain region- and sex-specific transcriptional profiles of microglia

**DOI:** 10.3389/fpsyt.2022.945548

**Published:** 2022-08-24

**Authors:** Kelly Barko, Micah Shelton, Xiangning Xue, Yvette Afriyie-Agyemang, Stephanie Puig, Zachary Freyberg, George C. Tseng, Ryan W. Logan, Marianne L. Seney

**Affiliations:** ^1^Translational Neuroscience Program, Department of Psychiatry, University of Pittsburgh School of Medicine, Pittsburgh, PA, United States; ^2^Department of Biostatistics, University of Pittsburgh School of Public Health, Pittsburgh, PA, United States; ^3^Center for Neuroscience, University of Pittsburgh, Pittsburgh, PA, United States; ^4^Department of Pharmacology and Experimental Therapeutics, Boston University School of Medicine, Boston, MA, United States; ^5^Center for Systems Neuroscience, Boston University, Boston, MA, United States; ^6^Department of Cell Biology, University of Pittsburgh, Pittsburgh, PA, United States; ^7^Genome Science Institute, Boston University School of Medicine, Boston, MA, United States

**Keywords:** microglia, RNA-sequencing, Tmem119, disease-associated microglia, sex difference

## Abstract

Microglia are resident macrophages of the brain, performing roles related to brain homeostasis, including modulation of synapses, trophic support, phagocytosis of apoptotic cells and debris, as well as brain protection and repair. Studies assessing morphological and transcriptional features of microglia found regional differences as well as sex differences in some investigated brain regions. However, markers used to isolate microglia in many previous studies are not expressed exclusively by microglia or cannot be used to identify and isolate microglia in all contexts. Here, fluorescent activated cell sorting was used to isolate cells expressing the microglia-specific marker TMEM119 from prefrontal cortex (PFC), striatum, and midbrain in mice. RNA-sequencing was used to assess the transcriptional profile of microglia, focusing on brain region and sex differences. We found striking brain region differences in microglia-specific transcript expression. Most notable was the distinct transcriptional profile of midbrain microglia, with enrichment for pathways related to immune function; these midbrain microglia exhibited a profile similar to disease-associated or immune-surveillant microglia. Transcripts more highly expressed in PFC isolated microglia were enriched for synapse-related pathways while microglia isolated from the striatum were enriched for pathways related to microtubule polymerization. We also found evidence for a gradient of expression of microglia-specific transcripts across the rostral-to-caudal axes of the brain, with microglia extracted from the striatum exhibiting a transcriptional profile intermediate between that of the PFC and midbrain. We also found sex differences in expression of microglia-specific transcripts in all 3 brain regions, with many selenium-related transcripts more highly expressed in females across brain regions. These results suggest that the transcriptional profile of microglia varies between brain regions under homeostatic conditions, suggesting that microglia perform diverse roles in different brain regions and even based on sex.

## Introduction

As the brain’s resident macrophage, microglia are instrumental to the regulation of parenchyma health, surveilling the local environment using a complex network of ramified processes through which they identify potential threats (i.e., cellular debris, microorganisms, misfolded proteins). In response to infection or injury, microglia rapidly change morphology to take on the classic activated ameboid form characterized by retracted, thickened processes and increased soma size. Activated microglia swarm the site of injury, release pro-inflammatory cytokines [i.e., interleukin (IL)-1β, IL-6, tumor necrosis factor (TNF)-α (1, 2)], and recruit peripheral macrophages to the site of injury. Microglia clear or engulf dead and dying cells (3) which prevents further damage caused by the release of cellular contents. Once the threat has been addressed, microglia release anti-inflammatory cytokines and growth factors to repair damage and restore homeostasis (1). Beyond their disease- and injury-associated functions, microglia play roles in shaping neuronal circuits during specific developmental periods, and evidence suggests that this process continues throughout the lifespan. In the healthy brain, microglia interact with and eliminate synapses and clear apoptotic neurons (3, 4), but crucially, they also induce synapse formation and regulate neurogenesis (5, 6). Microglia release synaptic factors (e.g., BDNF, glycine, L-serine) and prune synapses in an activity-dependent manner with implications for learning and behavior (4, 6–9).

Evidence from both preclinical and clinical studies reveal that microglia are heterogenous across brain regions in their density (10), morphology (11–14), gene expression (13–18), and proliferation (19–21). While not completely understood, the wide regional heterogeneity of microglia suggests they play distinct roles in specific regions of the brain. An early study by Lawson et al. demonstrated a greater than 5-fold variation in the density of microglial processes between regions (10). Frontal regions, including the cortex, striatum, and hippocampus, exhibit high levels of microglial ramification (11, 12), while microglia in hindbrain regions (i.e., cerebellum, brainstem) and regions which do not have a protective blood brain barrier (i.e., median eminence, circumventricular organs, subventricular zone) have low ramification tending more toward the activated ameboid morphology (10, 13, 14). The expression of molecular markers is region-specific. For instance, expression of the fractalkine receptor C-X3-C Motif Chemokine Receptor 1 (CX3CR1), a major component of the signaling pathway between microglia and neurons in the healthy brain, is highest in frontal regions and midbrain but comparatively low in hindbrain and circumventricular regions (18). The opposite pattern is true for phagocytic or immune activating genes, with, for instance, higher expression of markers associated with microglia reactivity in the blood brain barrier-lacking circumventricular organ of the mouse brain (13–17).

Further, evidence suggests that microglia may be phenotypically distinct between males and females (22–27). Brain sexual dimorphism is regulated by gene expression and hormonal surges during discrete developmental windows. By birth, microglia exhibit sex differences in number, morphology, and expression of activation markers/receptors. For instance, there are sex differences in levels of microglia with activated morphology during early postnatal development in several brain regions [e.g., in preoptic area, paraventricular nucleus, dentate gyrus, amygdala (26, 27)]. In these regions, sex differences in microglia number are dependent on steroid hormones produced during development; treating female mice with estradiol in the first two postnatal days produces the masculine pattern of microglia number and morphology (26). Evidence suggests sex differences in microglia phenotype as well. *Ex vivo*, microglia derived from male and female brains show divergent inflammatory signaling to lipopolysaccharide and estradiol (28). Evidence also suggests sex differences in the transcriptional profile of microglia [e.g., (23–25)], with female microglia showing a neuroprotective phenotype which is retained after transfer into male brains (23). There are also sex differences in morphology and transcript expression of microglia in the prefrontal cortex (25, 29), one of our regions of interest in the current study. Together, this prior evidence for sex differences in microglia motivates our current study to investigate the transcriptional profile of isolated microglia, focusing on whether any sex differences are consistent across brain regions.

Previous attempts to identify microglia in the CNS relied on morphology, relative marker expression as assessed by flow cytometry, or generating bone marrow chimeras [e.g., (10–16, 30–33) reviewed in (34)]. However, these approaches cannot be used to identify and isolate microglia in all contexts. Some commonly used markers are not cell type exclusive. For example, the commonly used marker for the fractalkine receptor CX3CR1 is also expressed by circulating monocytes and peripheral macrophages (35–37). Similarly, both microglial morphology and the expression of common microglial markers may change in response to disease or injury (e.g., expression of the purinergic receptor P2RY12 is lower in response to immune activation, while expression of CD68, a lysosomal-associated membrane protein, increases) (38–40). This adds an additional confounding factor to analyses which are aimed at generating a transcriptional profile across all microglia regardless of pathological state. Here, we make use of the microglia specific marker, transmembrane protein 119 (TMEM119), a robustly expressed cell-surface protein, to distinguish microglia from infiltrating macrophages (41). We used mice that conditionally expressed a fluorescent reporter only in TMEM119 expressing cells to sort microglia for RNA-sequencing (42). These mice enabled us to create transcriptomic profiles of purified microglia from male and female mice across the PFC, striatum, and midbrain to investigate potentially unique populations of microglia by sex and brain region. The three regions are reciprocally connected, functionally related, and activity across these regions underlies a multitude of complex behaviors. The direct connections between the dorsal and ventral striatum in the forebrain and the ventral tegmental area (VTA) and substantia nigra (SNc) in the midbrain represent a conserved dopaminergic circuit which is central to regulating movement, motivation, reinforcement, and learning (43–46). While the vast body of work has examined the ways that microglia in these regions respond to infection, disease, and degeneration, comparatively few groups have investigated the regional heterogeneity of this important cell type under homeostatic conditions, which we directly assess at the transcriptional level in the current study.

## Materials and methods

### Mice

Mice were group-housed (3–5 mice/cage) and maintained under standard conditions (12:12 h light/dark cycle; lights on 7 a.m.; 22 ± 1°; food and water *ad libitum*), in accordance with University of Pittsburgh Institutional Animal Care and Use Committee. Heterozygous Tmem119-2A-CreERT2 (Jackson Labs; RRID:IMSR_JAX:031820) female mice were crossed with a homozygous Ai14(RCL-tdTomato)-D (Jackson Labs; RRID:IMSR_JAX:007914; contains *loxP* flanked STOP cassette to prevent transcription of tdTomato reporter) male mouse to produce a Tmem119-2A-CreERT2/Ai14(RCL-tdTomato)-D mouse strain that allowed for conditional activation of the tdTomato reporter in TMEM119 labeled cells in the brain using tamoxifen. Adult mice heterozygous for Tmem119-2A-Cr eERT2 and Ai14(RCL-tdTomato)-D, 17–18 weeks in age were used for experimentation (*n* = 6 mice per sex). Mice were group-housed in 12-h light/dark with food and water *ad libitum*. Tamoxifen (Sigma−Aldrich, order no. 10540−29−1) solution preparation and administration were followed accordingly to instructions from The Jackson Laboratory, Bar Harbor, Maine^1^ (47, 48). Mice were administered 75 mg tamoxifen/kg body weight via intraperitoneal injection once every 24 h, for five consecutive days. Mice were sacrificed 10–21 days post final injection to allow for effective tamoxifen induction.

### Tissue extraction

**FIGURE 1:**
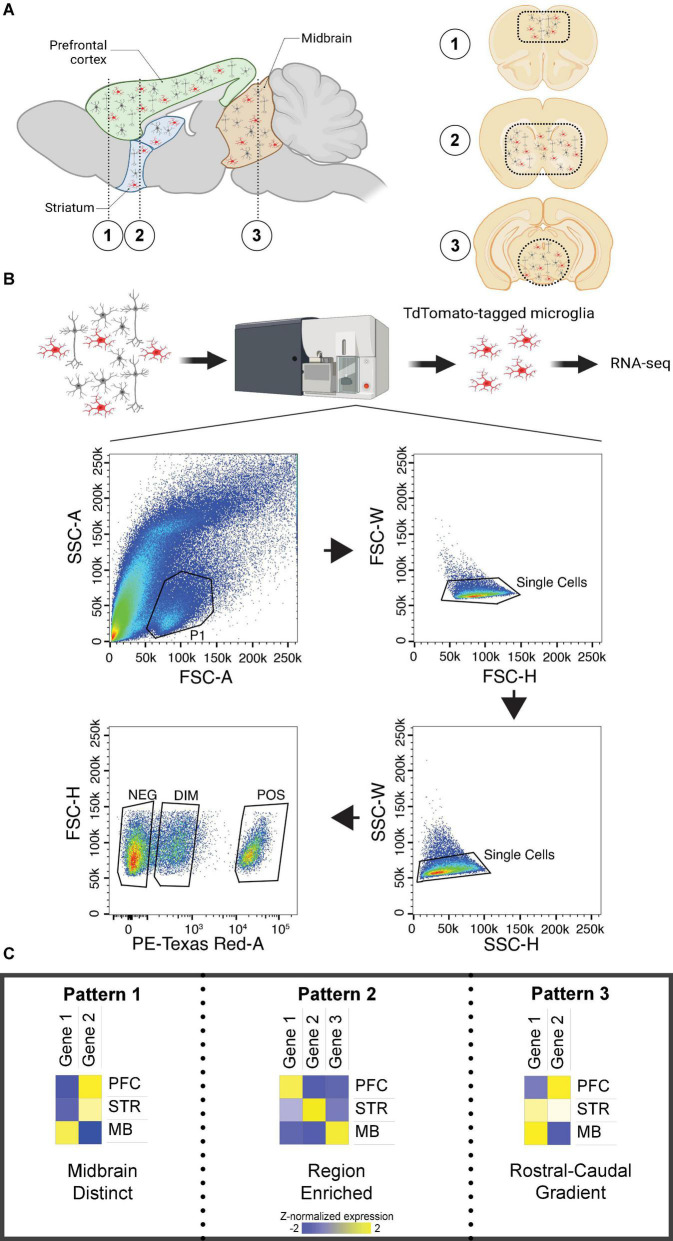
Brain regions investigated and patterns of differential expression across regions. **(A)** Left. Sagittal section of the mouse brain with prefrontal cortex (PFC), striatum (STR), and midbrain (MB) labeled. **(A)** Right. Coronal sections of the mouse brain corresponding to the numbered regions in the left sagittal section. Dashed lines indicate the approximate location of tissue isolation for each region. **(B)** Experimental design for microglia isolation with fluorescent-activated cell sorting, including the gating strategy. Only cells identified as Td-Tomato positive were sequenced. **(C)** Patterns of transcript expression across investigated brain regions. Pattern 1 was associated with distinct expression in the midbrain. Pattern 2 was associated with enrichment in either PFC or striatum. Pattern 3 was associated with a rostral-to-caudal gradient of expression. Created with BioRender.com.

Mice were sacrificed by live-cervical dislocation without anesthesia. Brains were extracted and rinsed with chilled 1X artificial cerebrospinal fluid (ACSF). Brain regions (prefrontal cortex; Bregma + 2.96 to + 1.42 mm, striatum; Bregma + 1.42 to –0.46 mm, and mid-brain; Bregma –2.88 to –3.88 mm) were separated by using a stainless-steel mouse brain matrix (1 mm) and single edge blades, kept on wet ice (Figure 1A). Sectioned tissue was transferred to designated 15 ml conical tubes filled with approximately 3 ml of chilled 1X ACSF.

### Tissue dissociation and preparation

Individual cell suspensions of harvested tissue for fluorescent activated cell sorting (FACS) were prepared by utilizing the following Miltenyl Biotec products: Adult Brain Dissociation Kit, mouse and rat (order no. 130-107-677), gentleMACS Octo Dissociator with Heaters (no. 130-096-427), gentleMACS C Tubes (no. 130-093-334), and MACS SmartStrainer (70 um) (no. 130-098-462). Cells were resuspended in 200 μL of 1X phosphate buffered saline (Gibco, order no. 70011-004) and transferred to designated 1.5 mL tubes for FACS. One sample from the PFC was lost during tissue dissociation due to a cracked tube, resulting in *N* = 5 male PFC samples.

### Fluorescent activated cell sorting

Individual cell suspensions were sorted by the Unified Flow Core FACS facility at the University of Pittsburgh. Cells containing the microglia marker of interest, Tmem119-2A-CreERT2, expressing Ai14(RCL-tdTomato)-D were sorted with a BD FACSAria II; cells were excited by a 532 nm laser and detected with 610/20 bandpass filter. The isolation and gating strategy is shown in Figure 1B; only positive cells were collected for RNA-seq. We confirmed using qPCR that positive cells exhibited high expression of the microglia-specific markers *Tmem119* and *Aif1*, and negligible expression of the neuronal marker *Rbfox2* (Supplementary Figure 1). Cells were sorted into designated 1.5 mL tubes with 250μL solution of Buffer RLT Plus (Qiagen, order no. 1030963) and 2-mercaptoethanol (MilliporeSigma, order no. 444203). Isolated microglia samples were stored at –80°C until further use. The mean number of isolated cells was 33,399 ± 4,272 (mean ± SEM; Supplementary Extended data 1).

### RNA sequencing

RNA extractions, cDNA generation, and library preparation were performed by the University of Pittsburgh Health Science Sequencing Core at the UPMC Children’s Hospital of Pittsburgh, Pittsburgh, PA, United States. RNA was extracted using the Qiagen RNeasy Plus Micro extraction kit (Qiagen:74034) following manufacturer’s instructions, including the use of DNA elimination columns. RNA was assessed for quality on an Agilent Fragment Analyzer 5300 using the High sensitivity RNA kit (Agilent: DNF-472-1000). The mean RNA integrity number (RIN) was 8.4, indicating excellent quality for RNA sequencing. 4.5 μl of RNA was used from each sample for cDNA generation using the Takara Smart-Seq HT kit (Takara: 634438) following manufacturer’s instructions, with 15 cycles of cDNA amplification. Smart-Seq cDNA was assessed for quality on an Agilent Fragment Analyzer 5300 using the High sensitivity NGS kit (Agilent: DNF-474-1000). All samples passed QC with full length cDNA (mean concentration of 12.9 ng/μl; primary peaks ∼2000 bp; absence of short length cDNA with bimodal peaks including second peak at ∼300 bp). Library preparation was performed using 1 ng of cDNA input with the Illumina Nextera XT kit (Illumina: FC-131-1096) and UDI indexes (Illumina: 20027215) added using 12 PCR cycles. Libraries were assessed using an Agilent High sensitivity NGS kit (Agilent: DNF-474-1000), and then normalized and pooled by calculating the nM concentration based on the fragment size (base pairs) and the concentration (ng/μl). Prior to sequencing, library pools were quantified by quantitative polymerase chain reaction (qPCR) on the LightCycler 480 using the KAPA qPCR quantification kit. Libraries were sequenced on a NovaSeq 6000 at UPMC Genome Center on an S2 100 cycle flow cell, 2 × 50 bp, for an average of ∼30 million reads per sample. Microglia-specific cell type markers (e.g., *Tmem119*, *P2ry12*, *Aif1*, *Itgam*) are highly expressed in our sorted cells compared to other cell type markers, indicating that our cell sorts are indeed effective at isolating microglia (Supplementary Figure 2).

### Data analysis

FastQC (version 0.11.9) was used to determine the per base sequence quality, with a mean score of 36 across samples. Paired-end reads were preprocessed, adapters removed using trimmomatic (version 0.38), and trimmed reads were mapped to Mus musculus Ensembl GRCm38 using HISAT2 (version 2.2.0), with a mean overall alignment rate of 92% across samples.

After mapping, the total 46,078 Ensembl transcripts were filtered to remove low expression transcripts. Specifically, we divide samples into subgroups by sex and brain region, then we keep only transcripts with at least one count per million (CPM) in at least one subgroup. After the filtering, 21,236 remained for DE analysis. RNA-seq data were analyzed using DESeq2 using brain region and sex as the main effects. Principal component analysis was performed using ggplot and the function prcomp. For brain region comparisons, we first determined if there was a main effect of region on microglial transcript expression (adjusted *p* < 0.05). If there was a main effect of brain region, we then performed two-group *post hoc* comparisons; transcripts with *post hoc p* < 0.05 and fold change > 1.2 were considered differentially expressed (DE). We identified 3 patterns of transcript expression, which we then probed in more detail: (1) distinct expression in the midbrain; (2) enrichment in either striatum or PFC; (3) gradients of expression (Figure 1C). We then determined which transcripts exhibited a main effect of sex (*p* < 0.05; fold change > 1.2). From the full interaction model, we then extracted data for sex differences within each brain region (*p* < 0.05; fold change > 1.2). Pathway over representation was assessed using Metascape, with expressed transcripts as background. Rank Rank hypergeometric overlap (RRHO) was used as a threshold-free approach to determine if there were similar patterns of sex differences across brain regions (49).

## Results

### Differential expression identifies brain region-specific transcriptional patterns

**FIGURE 2:**
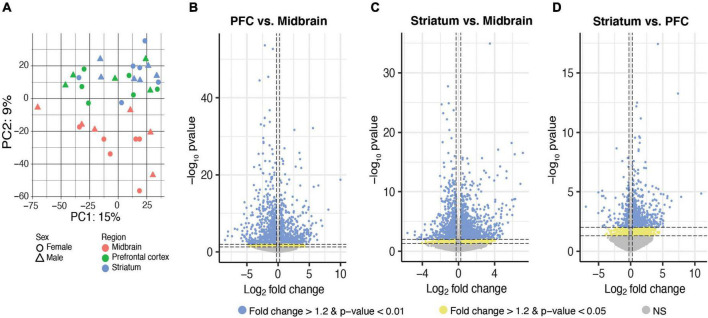
Differential expression of microglia-specific transcripts across brain regions. **(A)** Principal component (PC) analysis indicated distinct transcriptional profile in midbrain-isolated microglia compared to prefrontal cortex (PFC) and striatum. Log_2_FoldChange plotted relative to –log_10_pvalue by volcano plots for differentially expressed transcripts between PFC and midbrain **(B)**, striatum and midbrain **(C)**, and striatum and PFC **(D)**. Horizontal dashed lines represent p-value significance cutoffs of *p* < 0.01 and *p* < 0.05, while vertical dashed lines represent log_2_FC cutoffs of ≤ –0.26 or ≥ 0.26 (FC ≥ 1.2). Blue dots represent DE transcripts with *p* < 0.01 and FC ≥ 1.2. Yellow dots represent DE transcripts with *p* < 0.05 and FC ≥ 1.2.

We first determined whether there were brain region differences in transcript expression of isolated microglia by performing principal component analysis (PCA). A clear separation between transcripts enriched in the midbrain compared to those enriched in the PFC and striatum was observed; this effect is consistent for both males and females (Figure 2A). ANOVA detected 2,372 transcripts exhibiting a main effect of brain region (Supplementary Extended data 2) and *post hoc* two brain region comparisons revealed transcripts that were differentially expressed between: (1) PFC and midbrain (*p* < 0.05: 1368 transcripts; *p* < 0.01: 1226 transcripts; Figure 2B and Supplementary Extended data 3); (2) striatum and midbrain (*p* < 0.05: 1574 transcripts; *p* < 0.01: 1381 transcripts; Figure 2C and Supplementary Extended data 4); and (3) striatum and PFC (*p* < 0.05: 718 transcripts; *p* < 0.01: 486 transcripts; Figure 2D and Supplementary Extended data 5) (fold change for both *p*-value cutoffs > 1.2). These results suggest that microglia in the midbrain exhibit a distinct transcriptional profile compared to the PFC and striatum.

Three patterns of differential transcript expression were observed across brain regions in isolated microglia (Figure 1C). In line with the PCA, the first pattern was higher expression in the midbrain compared to PFC and striatum, with no difference between PFC and striatum [midbrain > (PFC = striatum)], or lower expression in the midbrain compared to PFC and striatum, with no difference between PFC and striatum [midbrain < (PFC = striatum)]. The second pattern included transcripts enriched in PFC or striatum. The final pattern included transcripts with a rostral-to-caudal gradient of expression, or the reverse of this gradient (i.e., caudal-to-rostral).

### Distinct transcriptional profile of microglia in the midbrain

**FIGURE 3:**
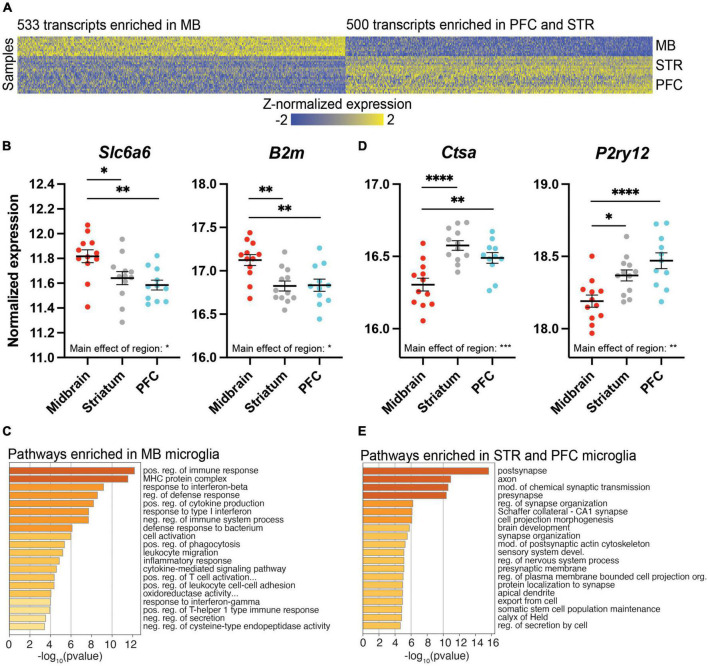
Differentially expressed (DE) transcripts that distinguish the midbrain (MB) from the prefrontal cortex (PFC) and striatum (STR). **(A)** Heatmap of DE transcripts between MB and both PFC and STR. The 533 transcripts more highly expressed in MB compared to both PFC and STR are indicated on the left of the heatmap, while the 500 transcripts expressed at lower levels in MB compared to both PFC and STR are indicated on the right of the heatmap. DE transcripts are plotted on the x-axis and individual subject samples on the y-axis. **(B)** *Slc6a6* and *B2m* fit the pattern of being more highly expressed in MB compared to PFC and STR. **(C)** Top pathways represented by transcripts expressed more highly in the midbrain compared to PFC and STR. **(D)** *Ctsa* and *P2ry12* fit the pattern of being expressed at lower levels in midbrain compared to PFC and STR. **(E)** Top pathways represented by transcripts expressed at lower levels in midbrain compared to PFC and STR. *, *p* < 0.05; **, *p* < 0.01; ***, *p* < 0.001; ****, *p* < 0.0001.

Given evidence for a distinct transcriptional profile in the midbrain compared to the PFC and striatum, we searched for transcripts exhibiting distinct expression in midbrain (Figure 3A). First, we considered transcripts with higher expression in the midbrain compared to PFC and striatum, with no difference between PFC and striatum [midbrain > (PFC = striatum)]; 533 transcripts fit this pattern, including the taurine transporter gene *Slc6a6* and beta-2 microglobulin (*B2m*; Figure 3B and Supplementary Extended data 6). Pathway analysis of the transcripts more highly expressed in midbrain, but equal expression in PFC and striatum [midbrain > (PFC = striatum)], identified pathways related to immune function, such as the MHC protein complex and positive regulation of immune response (Figure 3C and Supplementary Extended data 6).

We also considered the opposite expression profile in which transcripts were expressed at lower levels in the midbrain compared to PFC and striatum, with no difference between PFC and striatum [midbrain < (PFC = striatum)]; 500 transcripts fit this pattern, including the lysosomal enzyme cathepsin A (*Ctsa*) and the transcriptional regulator, *P2ry12*, which encodes purinergic receptor P2Y12 (Figures 3A,D and Supplementary Extended data 7). Overall, transcripts more lowly expressed in the midbrain with similar expression in PFC and striatum [midbrain < (PFC = striatum)] were involved in pre- and post-synapse function, and synapse organization (Figure 3E and Supplementary Extended data 7).

### Transcriptional profiles of microglia in the PFC and striatum

**FIGURE 4:**
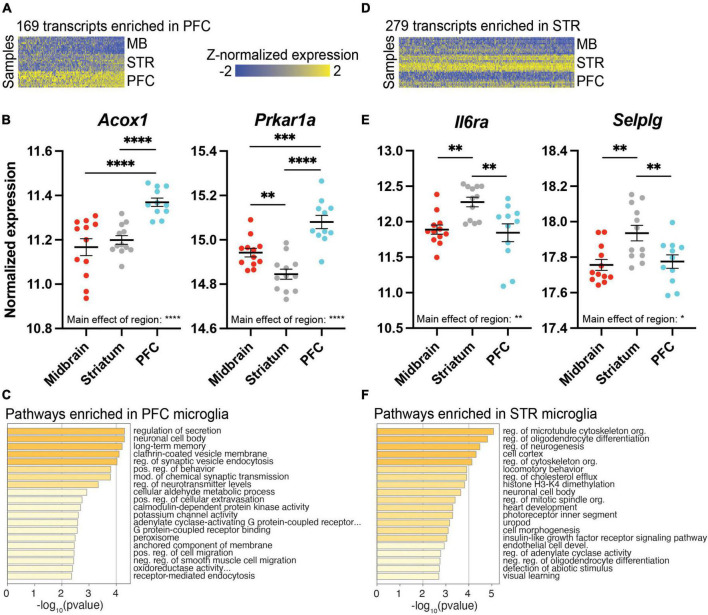
Differentially expressed (DE) transcripts enriched in prefrontal cortex (PFC) or striatum (STR). **(A)** Heatmap of 169 DE transcripts between PFC and both STR and midbrain (MB). DE transcripts are plotted on the x-axis and individual subject samples on the y-axis. **(B)** *Acox1* and *Prkar1a* fit the pattern of being more highly expressed in PFC compared to STR and MB. **(C)** Top pathways represented by transcripts expressed more highly in the PFC compared to STR and MB. **(D)** Heatmap of 279 transcripts enriched in STR compared to both PFC and MB. **(E)** *Il6ra* and *Selplg* fit the pattern of being more highly expressed in the STR compared to PFC and MB. **(F)** Top pathways represented by transcripts enriched in STR compared to PFC and MB. *, *p* < 0.05; **, *p* < 0.01; ***, *p* < 0.001; ****, *p* < 0.0001.

While the midbrain was most strikingly different from the PFC and striatum in terms of the transcriptional profile of microglia, we also identified transcripts enriched in PFC or striatum. We found 169 transcripts enriched in the PFC (Figure 4A and Supplementary Extended data 8), including *Acox1*, which codes for peroxisomal acyl-coenzyme A oxidase 1, and protein kinase cAMP-dependent type I regulatory subunit alpha (*Prkar1a*; Figure 4B). These PFC-enriched transcripts were associated with many synapse-related pathways, including long-term memory and regulation of synaptic vesicle endocytosis (Figure 4C and Supplementary Extended data 8). There were 279 transcripts enriched in the striatum compared to the PFC and midbrain (Figure 4D and Supplementary Extended data 9), including interleukin-6 receptor alpha (*Il6ra*) and the gene coding for P-selectin glycoprotein ligand 1 (*Selplg*) (Figure 4E). Transcripts enriched in striatal microglia were associated with pathways related to microtubules and the cytoskeleton (Figure 4F and Supplementary Extended data 9).

### Transcriptional profiling reveals expression gradients among microglia across brain regions

**FIGURE 5:**
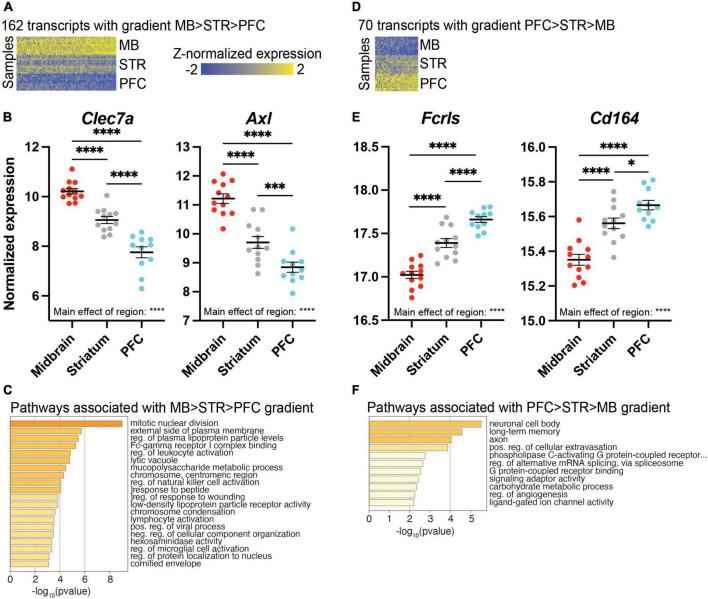
Differentially expressed (DE) transcripts exhibiting a gradient of expression across brain regions. **(A)** Heatmap of 162 DE transcripts with highest expression in the midbrain (MB), intermediate expression in the striatum (STR), and lowest expression in the prefrontal cortex (PFC). DE transcripts are plotted on the x-axis and individual subject samples on the y-axis. **(B)** *Clec7a* and *Axl* exhibit a gradient of expression of MB > STR > PFC. **(C)** Top pathways represented by transcripts exhibiting a gradient of expression of MB > STR > PFC. **(D)** Heatmap of 70 transcripts exhibiting highest expression in the PFC, intermediate expression in STR, and lowest expression in MB. **(E)** *Fcrls* and *Cd164* exhibit a gradient of expression of PFC > STR > MB. **(F)** Top pathways represented by transcripts exhibiting a gradient of expression of PFC > STR > MB. *, *p* < 0.05; ***, *p* < 0.001; ****, *p* < 0.0001.

We also identified transcripts that exhibited a gradient of expression across the rostral-to-caudal axis of the brain. These transcripts had highest expression in the midbrain, intermediate expression in the striatum, and lowest expression in the PFC; 162 transcripts fit this pattern (Figure 5A and Supplementary Extended data 10), including C-type lectin domain family 7 member 7 (*Clec7a*) and AXL receptor tyrosine kinase (*Axl*; Figure 5B). Pathway analysis revealed that these transcripts are involved in mitotic nuclear division and external side of plasma membrane (Figure 5C and Supplementary Extended data 10). We also considered the opposite gradient pattern, with transcripts that were most highly expressed in the PFC, with intermediate expression in the striatum, and lowest expression in the midbrain; 70 transcripts fit this pattern of expression (Figure 5D and Supplementary Extended data 11). The homeostatic microglia marker *Fcrls* which encodes the Fc receptor-like S, scavenger receptor, fits this pattern, as does the chemotaxis-related gene *Cd164* (Figure 5E). Pathway analysis indicated involvement in axons and long term memory (Figure 5F and Supplementary Extended data 11).

### Sex differences in transcript expression in microglia

**FIGURE 6:**
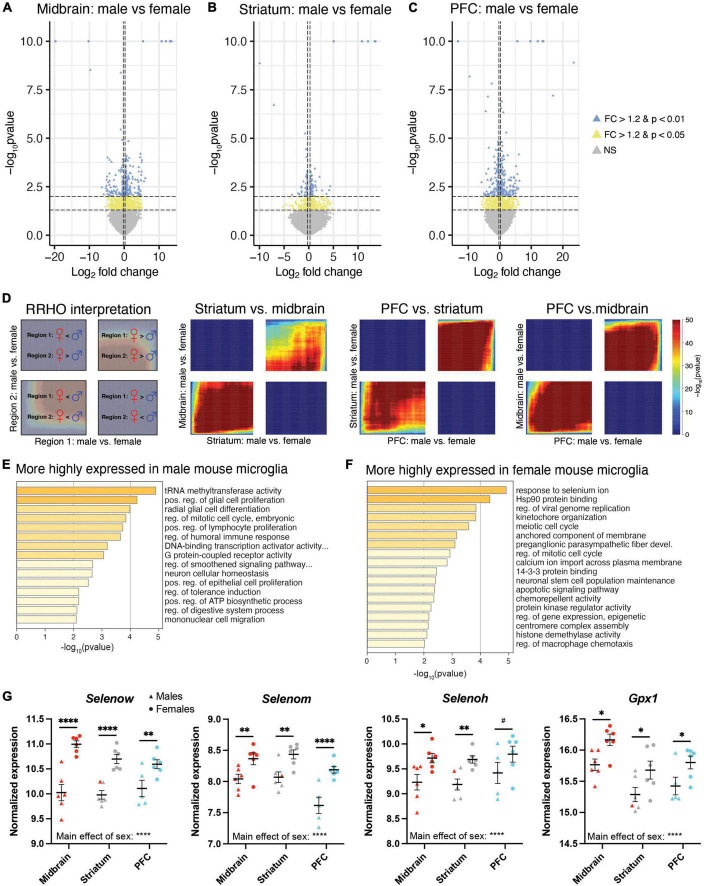
Differential expression of microglia-specific transcripts between males and females. Log_2_FoldChange plotted relative to –log_10_pvalue by volcano plots for differentially expressed (DE) transcripts between males and females in the midbrain (MB; **A**), striatum **(B)**, and prefrontal cortex (PFC; **C**). Horizontal dashed lines represent *p*-value significance cutoffs of *p* < 0.01 and *p* < 0.05, while vertical dashed lines represent log_2_FC cutoffs of ≤ –0.26 or ≥ 0.26 (FC ≥ 1.2). Blue triangles represent DE transcripts with *p* < 0.01 and FC ≥ 1.2. Yellow triangles represent DE transcripts with *p* < 0.05 and FC ≥ 1.2. **(D)** Rank rank hypergeometric overlap (RRHO) plots indicating high degree of overlap of DE transcripts between males and females across brain regions. The interpretation of RRHO plots is indicated on the left, followed by RRHO plots representing 2 brain region comparisons. Enrichment in the bottom left and top right quadrants indicates consistent sex differences across regions. **(E)** Top pathways associated with transcripts more highly expressed in male microglia. **(F)** Top pathways associated with transcripts more highly expressed in female microglia. **(G)** The selenium-related transcripts, *Selenow*, *Selenom*, *Selenoh*, and *Gpx1* were all more highly expressed in female microglia across all three brain regions. *, *p* < 0.05; **, *p* < 0.01; ****, *p* < 0.0001; ^#^, *p* < 0.1.

Although our PCA (Figure 2A) indicated that brain region explained most of the variance in transcript expression in microglia, previous studies suggest that microglia exhibit sex differences in morphology and gene expression (22, 23, 26–28). Given evidence that some sex differences in microglia are brain region specific, we first looked for sex differences in transcript expression within each brain region separately. In the midbrain, there were 924 DE transcripts by sex (male > female: 535 transcripts; female > male: 389 transcripts; Figure 6A and Supplementary Extended data 12). In the striatum, 454 transcripts were differentially expressed by sex (male > female: 288 transcripts; female > male: 166 transcripts; Figure 6B and Supplementary Extended data 13). In the PFC, there were 982 DE transcripts by sex (male > female: 534 transcripts; female > male: 448 transcripts; Figure 6C and Supplementary Extended data 14). As expected, many sex chromosome transcripts were among the top DE transcripts in all three brain regions (e.g., *Eif2s3y*, *Ddx3y*, *Uty*, *Kdm5d* more highly expressed in males; *Xist*, *Tsix* more highly expressed in females). Given the overlap in the top DE transcripts across brain regions, we more globally examined the overlap of transcripts that are DE by sex across regions using the threshold-free approach RRHO. Indeed, we found that the pattern of DE transcripts was quite similar between midbrain and striatum, between PFC and striatum, and between PFC and midbrain (Figure 6D). Thus, we next went back to our full model and determined that 891 transcripts exhibited a main effect of sex, with most of these transcripts being more highly expressed in males (male > female: 586 transcripts; female > male: 305 transcripts; Supplementary Extended Data 15). Pathway analysis indicated that transcripts enriched in male microglia across brain regions were associated with immune-related pathways (e.g., positive regulation of lymphocyte proliferation) and G protein-coupled receptor activity (Figure 6E and Supplementary Extended data 15). Transcripts more highly expressed in female microglia across brain regions were associated with response to selenium ion (Figure 6F and Supplementary Extended data 15), including *Selenow*, *Selenoh*, *Selenom*, and *Gpx1* (Figure 6G). Similar pathways were found when assessing each brain region separately (Supplementary Figure 3; Supplementary Extended datas 12–14).

## Discussion

### Summary of findings

Here we examined the transcriptional profile of microglia in three brain regions of treatment-naïve adult mice: the PFC, striatum, and midbrain. Our findings demonstrate that transcript expression between these regions differs substantially and follows one of 3 patterns. In the first and most common pattern, the transcriptional profile of midbrain microglia was distinct from the PFC and striatum. Our analysis identified many transcripts which were enriched in the midbrain with similar expression between PFC and striatum. Midbrain-enriched microglia were associated with pathways related to immune function, such as positive regulation of immune response, MHC protein complex, and response to interferon beta. We also identified transcripts with lower expression in the midbrain compared to the other two regions. These forebrain-enriched microglia were part of pathways related to synaptic function, including synapse organization and post synaptic genes. While the greatest difference in transcriptional profile was between the midbrain and forebrain, we also identified transcripts exhibiting other patterns of expression. In the second pattern, transcripts were enriched in either PFC or striatum compared to the other two regions. PFC-enriched transcripts were found in pathways associated with synapses, including regulation of synaptic vesicle endocytosis and modulation of chemical synaptic transmission. Transcripts enriched in striatal microglia were enriched for pathways associated with microtubules and cytoskeleton organization. In the third expression pattern, a subset of transcripts exhibited gradients in expression. Transcripts exhibiting a midbrain > striatum > PFC gradient were involved in mitotic nuclear division and external side of plasma membrane, as well as pathways associated with neuroinflammation. On the other hand, transcripts exhibiting a PFC > striatum > midbrain gradient were involved in axons and long-term memory. Finally, we found consistent sex differences in microglia-specific transcript expression across all three brain regions, with notable enrichment for selenium-related transcripts in female microglia.

### Brain region differences in the transcriptional profile of microglia

Though these three regions are related, we found that midbrain microglia were distinct from microglia in the PFC and striatum. Notably, several immune-related pathways were enriched in midbrain microglia compared to microglia isolated from the PFC or striatum. For instance, the taurine transporter gene, *Slc6a6*, is enriched in midbrain compared to PFC and striatum, and has previously been shown to be upregulated during M1 macrophage polarization (50). Additionally, *B2m*, a component of major histocompatibility complex (MHC) class 1 which we find to be more highly expressed in midbrain microglia, exhibits elevated expression in disease associated microglia [DAM (51, 52)]. Transcripts associated with homeostatic microglia, including the transcriptional regulator, *P2ry12* (53), were more lowly expressed in midbrain microglia. Our findings fall in line with previous studies of microglial regional heterogeneity in which others have demonstrated that *P2ry12* is higher in cortex and striatum, and comparatively lower in the midbrain (17, 34), while the expression of phagocytic and immune-activating genes was higher in midbrain compared to forebrain regions (16, 17, 34). The purinergic receptor P2Y12 is an important cell surface protein which microglia use to interact with other cell types. P2Y12 is responsible for sensing ATP in the environment released by both overactive neurons and by dead or dying neurons and initiates the movement of microglial processes toward the site of injury. P2Y12 signaling is thus necessary for not only mounting the microglial neuroinflammatory response (54, 55), but also for normal synaptic function and neurophysiology (56). Together, our findings support the conclusion that midbrain microglia exhibit a more immune-vigilant signature, while microglia in the forebrain are more homeostatic.

There were also transcripts enriched within the PFC or striatum as compared to the other two regions. PFC-enriched transcripts were enriched for synapse-related pathways. *Prkar1a* was enriched in PFC microglia, and this transcript has been shown to be elevated in surveillant microglia (57). Striatal-enriched microglia were associated with microtubule- and cytoskeleton-related pathways. *Il6ra* and *Selplg* were enriched in microglia isolated from the striatum; notably, *Selplg* plays a role in microglia’s ability to sense the environment (58). Together, these findings suggest midbrain microglia have a more disease-associated or immune-vigilant transcriptional profile, while cortical and striatal microglia have a transcriptional profile oriented toward remodeling synaptic and neuronal architecture as well as sensing the local environment. Because plasticity in these regions underlies learning and memory, it is possible that microglia in forebrain areas spend more time surveilling the local environment and performing functions related to synapse dynamics.

It is difficult to say with certainty what it means functionally for midbrain microglia to have a disease-associated transcriptional profile in treatment-naïve, healthy mice. However, we can look at other microglia with a similar transcriptional expression profile for clues. Previous work has demonstrated that microglia from regions with a more fenestrated BBB, such as the median eminence, the subventricular zone (SVZ), and circumventricular organs (CVOs), also have a disease-associated profile under normal homeostatic conditions. Microglia in regions with an incomplete BBB are characterized by downregulation of identity markers and upregulation of immune markers such as CD16/32 and CD86 (13–15, 31); however, there was no effect of brain region on these markers in our dataset. Morphologically, these microglia have shortened, thicker processes, a phenotype which is typically associated with an activated state (10, 13, 14). Other research has demonstrated that midbrain microglia have more sparse branching with smaller tissue coverage compared to striatal microglia (16). It is interesting to speculate that microglia in these regions with an incomplete BBB could be constitutively activated because they are exposed to more potential threats from the periphery without the protection afforded by a complete BBB. Indeed, systemic administration of lipopolysaccharide, which induces an inflammatory response, produces robust microglial proliferation, and an accompanying increase in microglial density, exclusively in CVOs and adjacent regions (59). For microglia in the midbrain, one could speculate that the local environment demands them to be in a similarly immune-vigilant state. However, it is important to note that pro-inflammatory cytokines released by microglia such as interleukin (IL)-1β, IL-6, and tumor necrosis factor (TNF)-α participate in the inflammatory process when faced with an immune challenge; however, under homeostatic conditions, these same cytokines regulate synaptic transmission and potentially work to regulate long-term synaptic plasticity (60). Along these lines, microglia in regions with an incomplete BBB express lower levels of the purinergic receptor P2Y12. Microglia in these regions extend their processes more slowly toward administered ATP than cortical microglia which express higher levels of P2Y12 (13). Consistently, midbrain microglia in our study also expressed lower levels of P2Y12. Functionally, this suggests that regulation of neurotransmission and synaptic functions by microglia could be region specific and regulated in part by markers typically associated with neuroinflammation.

### Microglia-specific transcript expression follows a gradient across regions

We found that there was a hierarchical pattern of expression across the brain regions that we assayed. While expression levels in the PFC and striatum were similar and expression in the midbrain was distinct, there were transcripts in which expression was highest in the PFC and lowest in the midbrain with an intermediate level of expression in the striatum. Transcripts which followed this pattern are associated with axons, long term memory, and G protein-coupled receptor binding. Two transcripts that fit this pattern are the homeostatic microglia marker *Fcrls* and the chemotaxis-related gene *Cd164* (61). There was also expression which followed the opposite pattern, with the highest expression in the midbrain and lowest in the PFC with the striatum being intermediate. Transcripts which fit this pattern were enriched for pathways such as mitotic nuclear division and external side of plasma membrane. Several neuroinflammatory pathways were identified in our gradient analysis, including regulation of leukocyte activation, regulation of natural killer cell activation, lymphocyte activation, and regulation of microglial cell activation, consistent with midbrain-enriched microglia exhibiting a more immune-surveillant phenotype. Consistent with these immune-related pathways, *Clec7a* and *Axl* exhibit this gradient of expression (midbrain > striatum > PFC), and these transcripts are more highly expressed in DAM (51, 52).

Others have demonstrated a similar rostro-caudal gradient in expression. Identity markers (i.e., CX3CR1, P2RY12) and immune-inhibitory genes such as *Sirpa* and *Cd206* are highly expressed in forebrain regions including the cortex, hippocampus, and striatum, and exhibit lower levels in midbrain and hindbrain regions such as VTA, cerebellum, and brainstem (15, 17, 34, 62). Phagocytic or immune activating genes show the opposite pattern of enrichment: high in hindbrain and midbrain structures but lowly expressed in forebrain regions (16, 17, 63). The gradient in transcript expression is concomitant with a gradient in microglial density and morphology. The density of microglia is high in cortex and hippocampus, intermediate in midbrain nuclei, and low in the hindbrain. The same pattern is repeated with regards to the ramification of microglial processes (10–12, 16). These rostral-caudal changes in microglia could reflect the increasing complexity of the dendritic arbor and a related increase in spine density as you move more rostral through the parenchyma (64).

Work by De Biase et al. suggests that microglial diversity may be more nuanced than just a rostral-to-caudal relationship. The authors focused on regional differences within the basal ganglia circuit nuclei. Like our work, they demonstrated that midbrain VTA microglia were distinct from cortical and striatal microglia. However, variation in cell density, process complexity, and lysosome content was largest between immediately adjacent midbrain nuclei. Substantia nigra pars reticulata (SNr) microglia exhibited a transcriptional profile distinct from substantia nigra pars compacta (SNc) and VTA microglia which were comparatively much more similar to NAc microglia (16). This indicates that while a rostro-caudal gradient in microglia transcriptional profiles exists across brain regions, local environments exist along the neuroaxis, and microglia respond to cues within those discrete areas. De Biase et al. further demonstrate that the ratio of microglia to neurons and other glial cell types, particularly astrocytes, changes in accordance with differences in microglial function and morphology; regional microglial phenotype was restored after pharmacologic ablation. These findings suggest that rather than epigenetic programing set during development, cell-extrinsic regulatory signals produce and maintain regional microglial identity in the adult organism. The midbrain contains other important structures, including perhaps most notably the dorsal raphe nuclei (DRN), which is responsible for serotonin synthesis within the brain. These serotonergic cells receive a range of brainstem inputs and project to an array of forebrain nuclei and play a crucial role in modulating complex behaviors (i.e., mood, reward, motivation, and learning). This nucleus consists of a diversity of cell types which contain multiple neurotransmitter types that are transcriptionally heterogenous (65–68). The periaqueductal gray (PAG) is another midbrain region relevant to autonomic function, motivation, defensive behavior, and pain modulation. Interestingly, evidence suggests that microglia within the PAG exhibit sex differences in levels of activation (female > male), and that female PAG microglia are more responsive to an immune challenge (69). Thus, the DRN and PAG represent discrete, local environments that may have distinct pools of microglia which might vary by sex or exhibit different transcriptional profiles compared to other brain regions. While we did not differentiate between subregions of the midbrain, such analyzes might be interesting in the future to discern whether microglia isolated from these discrete midbrain regions exhibit transcriptional differences.

Other research has corroborated this conclusion in both human and mouse. It has been consistently demonstrated that microglia express a composite transcriptional profile across regions which distinguishes them from other cell types such as peripheral macrophages, but which is expressed at different ratios within discrete structures. The maintenance of microglial phenotype depends on yet to be identified signals *in vivo*, and microglia lose their phenotype *ex vivo* and *in vitro* (15, 70–73). All these lines of evidence suggest that microglial regional heterogeneity depends on local signaling from other cell types which vary from region to region in terms of density, morphology, and function.

### Sex differences in transcript expression in microglia

While brain region explained most of the variance associated with the transcriptional profile of microglia, we also found sex differences in expression. This finding is consistent with a growing literature indicating sex differences in microglia (22, 23, 26–28).

The top pathway associated with transcripts more highly expressed in female microglia across all three brain regions was response to selenium ion (e.g., *Selenow*, *Selenoh*, *Selenom*, *Gpx1*). Gene products of these transcripts represent members of a class of Selenium (Se)-dependent proteins which participate in glucose metabolism and protect cells from oxidative stress by reducing reactive oxygen and nitrogen species (74, 75). Glutathione peroxidase 1 (GPX1) may play a further role in regulating the inflammatory response. For instance, overexpression of GPX1 results in fewer activated microglia after ischemic injury (76). There are many members of this diverse family with tissue-specific patterns of expression. Interestingly, expression of GPX1 and SelenoW is brain enriched. Spatial expression profiling indicates enrichment of SelenoW in 90% of brain regions assayed in the adult mouse (77). SelenoW has strong antioxidant properties and evidence suggests it plays a functional role in neuronal synapses and is highly expressed in the synaptic compartment (78, 79). In line with our findings, multiple lines of research demonstrate strong sex differences in selenoprotein expression and activity across domains including intracellular selenium metabolism, selenium recycling, absorption, and secretion. Selenium also directly influences the production of the sex hormone testosterone and, in return, sex hormones regulate selenium distribution and metabolism (74). For GPX1, female mice demonstrate greater efficiency in use of dietary selenite and higher expression of Gpx1 mRNA in peripheral tissue (80, 81). In humans, *Gpx1* SNPs also show sex differences leading to lower enzyme activity in males (82, 83). However, less is known about sex differences in activity and functional consequences of SelenoW expression. Further, existing research links GPX1 and SelenoW to risk for Alzheimer’s Disease (AD) (84). Selenium levels are significantly decreased in AD patients and carriers of the risk allele apolipoprotein E (ApoE4), making selenoproteins an intriguing site of inquiry (84). Both GPX1 and SelenoW are expressed in regions associated with the pathophysiology of AD and polymorphisms in human *Gpx1* have been significantly correlated with AD in two South American populations (85, 86). Chen et al. found SelenoW can form a disulfide bond to inhibit tau aggregation, which suggests this protein may play a crucial neuroprotective role (87). Microglia are directly linked to the pathophysiology of AD; activated microglia respond to the buildup of Aβ plaques, and the overactivation of microglia may lead to the pathological loss of synapses in the disorder. Outside of *APOE*, the majority of AD-associated risk loci are expressed exclusively or preferentially by microglia (88). It is interesting to speculate that sex differences in selenoproteins, possibly even within microglia, might contribute to sex differences in AD.

While we see mostly consistent sex differences in the microglia transcriptional profile across the PFC, striatum, and midbrain, there are some differences. Notably, we find that many synapse-related transcripts and associated pathways (e.g., “glutamatergic synapse” and “GABA receptor binding”) are more highly expressed in females in only the midbrain. Interestingly, a previous study by Guneykaya et al., examined sex differences in microglia transcript expression and identified “GABA and Glutamate receptor activity” associated with transcripts more highly expressed in females (25). Guneykaya et al., also found that male microglia are enriched for pathways associated with transcriptional activity in the cortex, similar to our findings in the PFC. We also found that across brain regions, male microglia exhibit a more inflammatory profile. This result is consistent with a previous study which examined the transcriptional profile of microglia isolated from the whole mouse brain (23). Overall, our results associated with sex differences in microglia transcript expression are consistent with previous studies.

### Limitations

One limitation to the current study is that we did not assess phase of estrous cycle in female mice at the time of sacrifice, which would have given us insight into whether levels of circulating ovarian hormones might influence transcript expression in females. Given our findings for sex differences in expression of microglia-specific transcripts, in future studies, it will be important to determine whether circulating gonadal hormones drive these sex differences. However, a previous study reported that phase of estrous cycle did not influence microglia-specific gene expression in the hippocampus (24). Future studies will also use similar methodology to probe for sex differences in brain regions more traditionally defined as being sexually dimorphic (e.g., hypothalamus). Another limitation is that the transgenic strain we used required tamoxifen injections to drive Cre expression. Tamoxifen can act as a potent estrogen receptor-alpha agonist and antagonist, and administration of tamoxifen may disrupt cyclicity in females [e.g., (89–93)]. Thus, it is possible that tamoxifen influenced some of our findings related to sex differences. However, we waited 10–21 days after tamoxifen injection to sacrifice mice, reducing the possibility for acute effects of tamoxifen on microglia-specific transcript expression. Further, evidence suggests that tamoxifen used for Cre-induction does not cause long-term effects or sex differential responses in the brain transcriptome (94). We report findings related to transcript expression within isolated microglia; thus, it is unclear if associated proteins will exhibit similar patterns. Future studies will assess protein levels of identified transcripts within microglia. Another limitation is that we might have missed a population of TMEM119-negative microglia, which could not be determined using the method employed here. Finally, it is likely that several factors contribute to inform regional phenotype, which cannot be divorced from each other. It is hard to disentangle the effects of local cues in the environment from putative identity differences inherent to microglia within different compartments. Factors from the local environment (e.g., from neurons, glia, as well as infiltrating blood-derived macrophages) all interact with microglia, which may contribute to regional heterogeneity. Indeed, there are regional differences in the density, relative cell ratio, and function of these other cell types which creates unique signaling milieus within discrete local environs to which microglia respond.

## Conclusion

Here, we find that the transcriptional profile of isolated microglia differs based on brain region and sex, even under homeostatic conditions. Future studies will use similar methodology to assess whether these transcriptional profiles shift when mice are exposed to stress, with a specific focus on whether males and females exhibit similar alterations. Microglia have in the past been thought of as a homogenous cell type which was alternatively quiescent during homeostasis and activated when the parenchyma was faced with threat or neurodegeneration. However, microglia play several roles in the healthy organism as well, and evidence suggests that there are several subtypes of microglia which can be distinguished by the transcripts they express. These putative subtypes can be found in different ratios within and across regions and may perform specific functions in response to stimuli (73). Indeed, our findings support the hypothesis that microglia perform diverse functions based on both brain region and sex.

## Data availability statement

Raw and processed RNA-sequencing data have been deposited into the NCBI Gene Expression Omnibus database (GEO; GSE203553).

## Ethics statement

The animal study was reviewed and approved by University of Pittsburgh Institutional Animal Care and Use Committee.

## Author contributions

MLS, RL, and ZF designed and coordinated the study. KB and SP obtained and processed samples using FACS sorting. XX, MLS, KB, GT, and YA-A conducted statistical analyzes and data interpretation. MS, YA-A, KB, and MLS drafted the manuscript. All authors contributed to the article and approved the submitted version.

## Footnotes

1
https://www.jax.org/research-and-faculty/resources/cre-repository/tamoxifen

## Supplementary material

Supplementary Extended Data 1 Mean number of isolated microglia across brain regions.

Supplementary Extended Data 2 Microglia-specific transcripts exhibiting a main effect of brain region.

Supplementary Extended Data 3 Microglia-specific transcripts exhibiting significant differences between prefrontal cortex and midbrain in *post hoc* analyzes.

Supplementary Extended Data 4 Microglia-specific transcripts exhibiting significant differences between striatum and midbrain in *post hoc* analyzes.

Supplementary Extended Data 5 Microglia-specific transcripts exhibiting significant differences between striatum and prefrontal cortex in *post hoc* analyzes.

Supplementary Extended Data 6 Microglia-specific transcripts with enrichment in midbrain (MB) compared to both prefrontal cortex (PFC) and striatum (STR), with no difference between prefrontal cortex and striatum [MB > (PFC = STR)].

Supplementary Extended Data 7 Microglia-specific transcripts with lower expression in midbrain (MB) compared to both prefrontal cortex (PFC) and striatum (STR), with no difference between prefrontal cortex and striatum [MB < (PFC = STR)].

Supplementary Extended Data 8 Microglia-specific transcripts with enrichment in prefrontal cortex compared to both striatum and midbrain.

Supplementary Extended Data 9 Microglia-specific transcripts with enrichment in striatum compared to both prefrontal cortex and midbrain.

Supplementary Extended Data 10 Microglia-specific transcripts with highest expression in midbrain (MB), intermediate expression in striatum (STR), and lowest expression in prefrontal cortex (PFC) [MB > STR > PFC].

Supplementary Extended Data 11 Microglia-specific transcripts with lowest expression in midbrain (MB), intermediate expression in striatum (STR), and highest expression in prefrontal cortex (PFC) [MB < STR < PFC].

Supplementary Extended Data 12 Microglia-specific transcripts exhibiting a sex difference in expression in the midbrain.

Supplementary Extended Data 13 Microglia-specific transcripts exhibiting a sex difference in expression in the striatum.

Supplementary Extended Data 14 Microglia-specific transcripts exhibiting a sex difference in expression in the prefrontal cortex.

Supplementary Extended Data 15 Microglia-specific transcripts exhibiting a main effect of sex across brain regions.
