# Supplementary material for: Brain region- and sex-specific transcriptional profiles of microglia
Source: Front Psychiatry. 2022 Aug 24;13:945548. doi: 10.3389/fpsyt.2022.945548 (PMC9448907; doi:10.3389/fpsyt.2022.945548)
Supplement: Supplementary file 1 [file Data_Sheet_1.docx]

Supplementary Material

Brain region- and sex-specific transcriptional profiles of microglia

Kelly Barko^1,#^, Micah Shelton^1,#^, Xiangning Xue^2^, Yvette Afriyie-Agyemang^1,3^, Stephanie Puig^4,5^, Zachary Freyberg^1,3,6^, George C. Tseng^2^, Ryan W. Logan^4,5,7^, Marianne L. Seney^1,3,*^

^1^Translational Neuroscience Program, Department of Psychiatry, University of Pittsburgh School of Medicine, Pittsburgh, PA, USA

^2^Department of Biostatistics, University of Pittsburgh School of Medicine, Pittsburgh, PA, USA

^3^Center for Neuroscience, University of Pittsburgh, Pittsburgh, PA USA

^4^Department of Pharmacology and Experimental Therapeutics, Boston University School of Medicine, Boston, MA 02118, USA

^5^Center for Systems Neuroscience, Boston University, Boston, MA 02118, USA

^6^Department of Cell Biology, University of Pittsburgh, Pittsburgh, PA 15213, USA

^7^Genome Science Institute, Boston University School of Medicine, Boston, MA 02118, USA

^#^Equal contribution

***Correspondence:**Marianne L. Seney, PhD

seneyml@upmc.edu

Keywords: microglia, RNA-sequencing, Tmem119, disease-associated microglia, sex difference.

1. **Supplementary Figures**


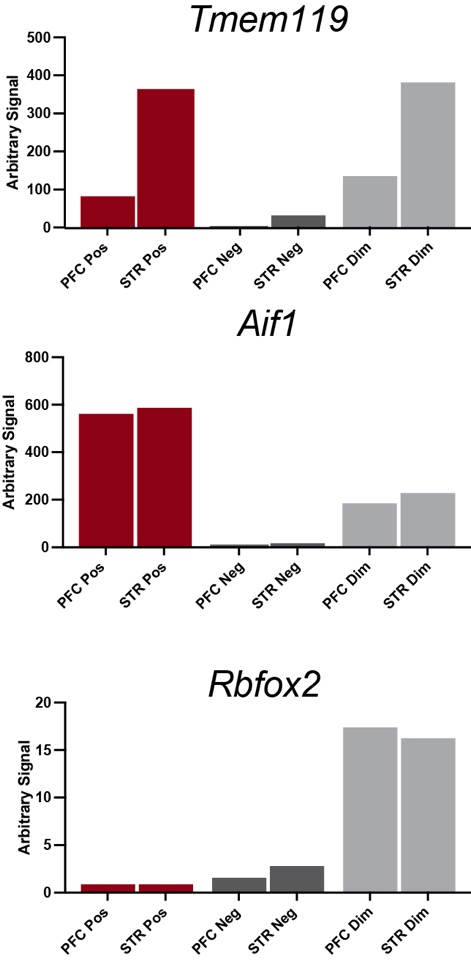


**Supplementary Figure 1. Enrichment for microglia-specific markers in isolated cells.** Quantitative polymerase chain reaction using BioRad mouse primers confirm that positive (Pos) cells isolated with our fluorescent activated cell sorting (FACS) gating strategy exhibit high levels of the microglia-specific markers *Tmem119* and *Aif1*, and negligible expression of the neuronal marker *Rbfox2*. Negative (Neg) cells exhibited negligible expression of *Tmem119*, *Aif1*, and *Rbfox2*. Dim cells exhibited expression of *Tmem119*, *Aif1*, and *Rbfox2*, suggesting that while they express microglia markers, these cell isolates might have contamination from neurons. Thus, we moved forward with performing RNA-sequencing on only positive cells.


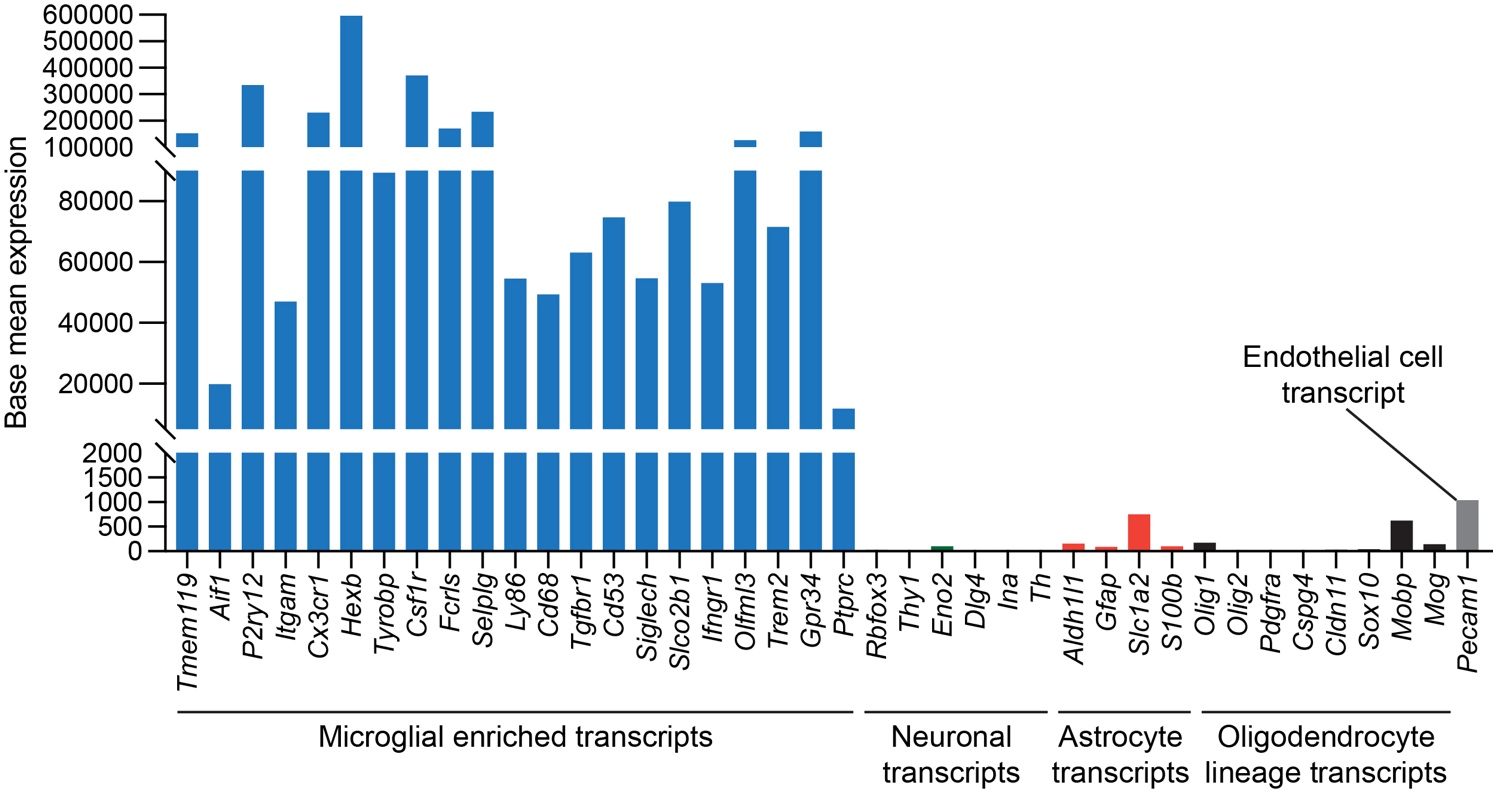


**Supplementary Figure 2. Enrichment for microglia-specific markers in sequenced pool of cells.** Investigation of base mean expression of cell type-specific markers confirmed that isolated cells were indeed microglia. Cells exhibited high expression of microglia-specific markers, negligible expression of astrocyte-specific markers, negligible expression of oligodendrocyte lineage-specific markers, negligible expression of neuron-specific markers, and low expression of an endothelial cell-specific marker.

**
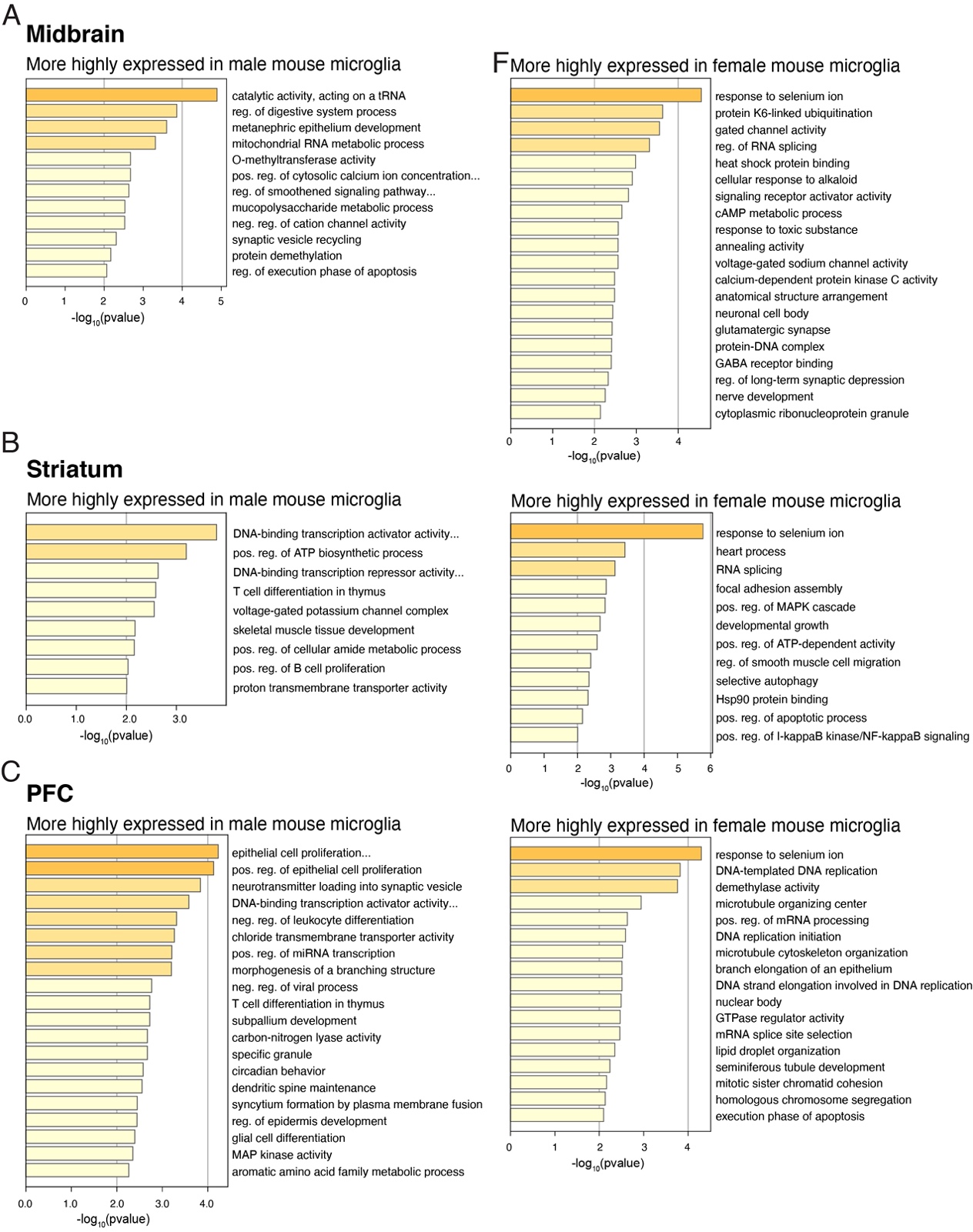
**

**Supplementary Figure 3. Top pathways associated with transcripts exhibiting sex differences within midbrain (A), striatum (B), and prefrontal cortex (PFC; C).**
